# Flagella, Type I Fimbriae and Curli of Uropathogenic *Escherichia coli* Promote the Release of Proinflammatory Cytokines in a Coculture System

**DOI:** 10.3390/microorganisms9112233

**Published:** 2021-10-27

**Authors:** Rubí Vega-Hernández, Sara A. Ochoa, Ricardo Valle-Rios, Gustavo A. Jaimes-Ortega, José Arellano-Galindo, Gerardo Aparicio-Ozores, José Antonio Ibarra, Rigoberto Hernández-Castro, Ariadnna Cruz-Córdova, Juan Xicohtencatl-Cortes

**Affiliations:** 1 Laboratorio de Investigación en Bacteriología Intestinal, Unidad de Investigación en Enfermedades Infecciosas, Hospital Infantil de México Federico Gómez, 06720 Ciudad de México, Mexico; rubivega95@gmail.com (R.V.-H.); saraariadnah@hotmail.com (S.A.O.); 2 Posgrado en Biomedicina y Biotecnología Molecular, Escuela Nacional de Ciencias Biológicas, Instituto Politécnico Nacional, 09340 Ciudad de México, Mexico; 3 Unidad Universitaria de Investigación en Cáncer e Inmunología, División de Investigación, Facultad de Medicina, Universidad Nacional Autónoma de México, 04510 Ciudad de México, Mexico; vallerios@unam.mx (R.V.-R.); gelet321@gmail.com (G.A.J.-O.); 4 Unidadde Investigación en Inmunología y Proteómica, Hospital Infantil de México Federico Gómez, 06720 Ciudad de México, Mexico; 5 Posgrado en Biología Experimental, Departamento de Ciencias Biológicas y de la Salud, Universidad Autónoma Metropolitana, 09340 Ciudad de México, Mexico; 6 Laboratorio de Virología Clínica y Experimental, Unidad de Investigación en Enfermedades Infecciosas, Hospital Infantil de Mexico Federico Gómez, 06720 Ciudad de México, Mexico; josearellanogalindo@yahoo.com.mx; 7 Departamento de Microbiología, Escuela Nacional de Ciencias Biológicas, Instituto Politécnico Nacional, 11340 Ciudad de México, Mexico; gaparici@hotmail.com (G.A.-O.); jaig19@gmail.com (J.A.I.); 8 Departamento de Ecología de Agentes Patógenos, Hospital General Dr. Manuel Gea González, 4800 Ciudad de México, Mexico; rigo37@gmail.com

**Keywords:** UPEC, adherence, fimbriae, cytokines, coculture

## Abstract

Background. Urinary tract infections (UTIs) are a public health problem in Mexico, and uropathogenic *Escherichia coli* (UPEC) is one of the main etiological agents. Flagella, type I fimbriae, and curli promote the ability of these bacteria to successfully colonize its host. Aim. This study aimed to determine whether flagella-, type I fimbriae-, and curli-expressing UPEC induces the release of proinflammatory cytokines in an established coculture system. Methods. The *fliC*, *fimH*, and *csgA* genes by UPEC strain were disrupted by allelic replacement. Flagella, type I fimbriae, and curli were visualized by transmission electron microscopy (TEM). HTB-5 (upper chamber) and HMC-1 (lower chamber) cells cocultured in Transwell^®^ plates were infected with these UPEC strains and purified proteins. There was adherence to HTB-5 cells treated with different UPEC strains and they were quantified as colony-forming units (CFU)/mL. Results. High concentrations of IL-6 and IL-8 were induced by the FimH and FliC proteins; however, these cytokines were detected in low concentrations in presence of CsgA. Compared with UPEC CFT073, CFT073Δ*fimH*, CFT073Δ*fimH*Δ*fliC*, and CFT073Δ*csgA*Δ*fimH* strains significantly reduced the adherence to HTB-5 cells. Conclusion. The FimH and FliC proteins are involved in IL-6 and IL-8 release in a coculture model of HTB-5 and HMC-1 cells.

## 1. Introduction

Urinary tract infections (UTIs) are one of the main causes of morbidity, affecting millions of people each year worldwide. UTIs mainly affect women; approximately 40% to 60% of adult women will have at least one UTI in their lifetime, and 28% to 48% of affected women will have a recurrent UTI in the same year [1,2,3,4]. It is known that the prevalence of UTIs increases with age and that 20% of women over 65 years old will experience a UTI episode [5]. In the Mexican population, UTIs are the third most common infection after respiratory and gastrointestinal infections, according to the National Epidemiological Surveillance System (SINAVE). Symptomatic UTIs are classified according to the severity; the lower UTIs are caused by bacteria in the bladder and trigger cystitis, while upper UTIs include infection of the kidney, which results in pyelonephritis [4,6,7]. UTIs can be uncomplicated (affecting healthy individuals without structural and/or functional urinary tract abnormalities) or complicated (affecting individuals with functional and/or structural urinary tract abnormalities) [8,9,10,11,12].

Uropathogenic *Escherichia coli* (UPEC) is the main causal agent of community- (80 to 90%) and hospital-acquired (30 to 50%) UTIs, which stands for more than half of all uncomplicated UTIs [4,13,14,15]. Recently, clinical strains of UPEC multidrug-resistant (MDR), extensivelydrug-resistant (XDR), and the prevalence of extended-spectrum beta-lactamase (ESBL)-producing infection bacteria were related with increases in the use of antibiotics. Furthermore, UPEC strains carry different classes of integrons (I and II) that have been associated with different phylogenetic groups (A, B2, and D), in addition to expressing toxins (*tosA* gene) and several virulence genes [16,17,18,19,20]. Moreover, UPEC O25-ST131 was characterized as an MDR strain that is highly genetically diverse, belongs to phylogenetic group B2, and expresses many virulence genes [18].

Virulence genes encode fimbrial adhesins (type I, P, F1C, S, and curli fimbriae), nonfimbrial adhesin (TosA), flagella, iron acquisition systems, polysaccharide capsule, biofilm production, and toxins (hemolysin), which are essential structures that contribute significantly to UPEC pathogenesis [16,21,22,23]. The specific virulence factors expressed by UPEC allow it to establish and maintain colonization and circumvent host defense mechanisms [24]. FimH (type I fimbriae), PapG variants (P fimbriae), and CsgA (curli fimbriae) adhesins are widely distributed in clinical UPEC strains from pediatric patients with complicated UTIs [25]. These fimbriae, which are located on the bacterial cell surface, aid in adhesion to the host cell surface, tissue invasion, biofilm formation, and cytokine induction [26]. Preliminary studies have suggested that the expression of type I fimbriae contributes to the colonization of UPEC in the bladder epithelium and that P and curli fimbriae participate in colonization when bacteria ascend to infect the kidney [24,27]. Recently, it was demonstrated that curli fimbriae enhance colonization of UPEC in the urinary tract in a C57BL/6 mouse infection model. Highly stable fusion proteins with FimH, CsgA, and PapG adhesins show antigenic properties and induce cytokine release as well as the production of antibodies against these proteins [24].

Several coculture models, including epithelial cell types cultured with neutrophils, eosinophils, monocytes, and lymphocytes, have been described [28]. UPEC type I fimbriae mainly triggers the secretion of certain cytokines, such as interleukin (IL)-6, Macrophage Inflammatory Protein-2 (MIP-2), IL-12, IL-18, and tumor necrosis factor-alpha (TNF-α), in a UTI mouse model [29]. Other studies have reported that Toll-like receptor (TLR)5, a member of the FliC pathway, triggers rapid IL-10 synthesis in the bladder and is a potential immune modulator that may play a role in the treatment or prevention of UPEC-mediated UTIs [30]. In contrast, the expression of type I fimbriae and bacterial binding to its host is not required for IL-10 release in a human bladder cell/monocyte mixed coculture system [28]. This study aimed to establish a coculture model using bladder epithelial cells (HTB-5) and human mast cells (HMC-1 cells) and evaluate whether heteropolymeric structures such as flagella, type I fimbriae, and curli fimbriae promote the release of pro- and anti-inflammatory cytokines during infection.

## 2. Materials and Methods

### 2.1. Bacterial Strains and Growth Conditions

All the bacterial strains and plasmids used in this study are detailed in Table 1. UPEC strain CFT073 was cultured on Luria-Bertani (LB) and MacConkey agar and incubated for 24 h at 37 °C. The *fliC*, *fimH*, and *csgA* genes were disrupted in this strain of UPEC. Variants of UPEC strain CFT073 in which different genes were disrupted were cultured on LB agar supplemented with ampicillin (Amp, 100 µg/mL), kanamycin (Km, 50 µg/mL) and/or chloramphenicol (Cm, 25 µg/mL) as required.

### 2.2. Design and Synthesis of Primers for Gene Disruption

Primers for mutation and verification of the *fliC*, *fimH,* and *csgA* genes were designed according to the genome sequence of UPEC strain CFT073 with accession number AE014075.1 (National Center for Biotechnological Information; NCBI). Primers for mutation, which contained 70 or 80 bp, including 50 or 60 nucleotides identical to the sequences flanking the 5′ ends of the mutated gene and 20 nucleotides that hybridized with the sequences of the 3′ ends of the plasmid pKD3 (Cm^R^) or pKD4 (Km^R^) (Table 2), which are flanked by FRT sequences recognized by FLP recombinase, were designed and synthesized [29]. PCR was conducted with PFUX polymerase (Jena Bioscience, Jena, Germany), and the products were purified using a Zymoclean Gel DNA Recovery Kit (ZymoResearch, Irvine, CA, USA).

### 2.3. Generation and Verification of Isogenic Mutants

The *fliC*, *fimH*, and *csgA* genes of UPEC strain CFT073 were disrupted as described by Datsenko and Wanner [31]. UPEC strain CFT073 was cultured in LB broth at 37 °C overnight, centrifuged, washed three times, and transformed with the pKD46 plasmid. Shocked cells were added to 1 mL LB broth and incubated for 2 h at 30 °C, and then one-half of the cells were spread on agar for the selection of ampicillin transformants. Then, these transformed cells were grown at 30 °C with constant shaking at an OD600 of 0.6 in 20 mL LB with ampicillin (100 µg/mL) and L-arabinose (1 mM) to induce red recombinase expression. The cells were transformed with the DNA products obtained from the gene of interest by endpoint PCR. The transformed colonies were recovered and selected after culturing them at 37 °C on LB agar plates supplemented with Km (50 µg/mL) and/or Cm (25 µg/mL).

Disruption of single genes (Δ*fliC*, Δ*fimH*, and Δ*csgA*) and double genes (Δ*fimH*Δ*fliC*, Δ*csgA*Δ*fimH*, and Δ*csgA*Δ*fliC*) was confirmed by PCR using primers corresponding to the region 100 bp upstream and 100 bp downstream of the ORF of the mutated genes (Table 3). Briefly, the concentrations of the reagents were adjusted to achieve a final volume of 12 µL comprising 6.25 µL of Master Mix^®^ (Promega, Woods Hollow Road, Madison, WI, USA), 1.5 µL of 1 µM each primer (forward and reverse), 0.75 µL of nuclease-free water, and 2 µL of the bacterial suspension. Amplification of each gene was performed with a Veriti 96-well thermal cycler (Applied Biosystems^®^, Lincoln Centre Drive Foster City, CA, USA) according to the specific hybridization temperature (Table 3). The *fliC* (1923 bp), *fimH* (1237 bp), and *csgA* (789 bp) of UPEC strain CFT073 were amplified as positive controls. The products obtained by PCR were separated on 1.5% agarose gels, stained with ethidium bromide, and visualized on a UV transilluminator.

### 2.4. Transmission Electron Microscopy and Protein Purification

Copper grids containing 300 quadrants (Electron Microscopy Sciences^®^, Hatfield, PA, USA) were covered with formvar (Sigma-Aldrich^®^, Westport, CT, USA) to visualize flagella, type I fimbriae, and curli fimbriae in UPEC strain CFT073. To promote curli expression, the strains were cultivated in yeast extract casamino acids (YESCA) medium supplemented with 4% dimethyl sulfoxide (DMSO) at 26 °C. To promote type I fimbriae expression, the bacteria were cultured on LB agar medium supplemented with dextrose (1 g/L) at 37 °C, and to promote flagella expression, the bacteria were cultured on 0.3% semisolid LB agar. Briefly, the formvar grids were incubated with 50 µL of each of the bacterial cultures for 5 min, the excess was removed, and the grids were washed with sterile water. Then, 50 µL of 1% phosphotungstic acid (PTA) was added for 5 min. Finally, the PTA was removed, and the samples were visualized by transmission electron microscopy (TEM) (Jeol Microscope Mod. JEM 1010).

Conversely, the purified FimH and CsgA proteins were made according to Luna-Pineda et al. (2016). For FliC, UPEC CFT073 was plated on 1% LB agar overnight at 37 °C. Bacteria were harvested in PBS, gently mechanically shaken for 10 min, and centrifuged at 500× *g* for 5 min. The bacterial pellet was discarded, and the supernatant was centrifuged again 1500× *g* for 10 min. Finally, the bacterial package was resuspended in 2 mL of PBS, which was subjected to 12% sodium dodecyl sulfate polyacrylamide gel electrophoresis (SDS-PAGE), and was visualized by Coomassie staining.

### 2.5. Standardization of Cultured TCCSUP (HTB-5™) Human Bladder Cells and HMC-1 Human Mast Cells

Human mast cells (HMC-1 cells, SCC062, Merck Millipore) were cultured in Roswell Park Memorial Institute (RPMI) 1640 medium (ATCC^®^, Manassas, VA, USA) in 24-well plates at 37 °C. Suspended cells were infected with UPEC strain CFT073 previously cultivated in LB medium at 37 °C at a multiplicity of infection (MOI) of 1:10. The infected cells were incubated for 3 to 5 h at 37 °C in 5% CO_2_. At the time of infection, cell viability was quantified employing the trypan blue exclusion method. The infected HCM-1 cells were collected from each well, centrifuged at 500× *g* for 1 min. The supernatants were frozen at −70 °C for quantification of cytokine levels. The infected cells were washed 3 times with phosphate-buffered saline solution (PBS) and treated with 1 mL of 0.1% Triton X-100 for 5 min. To quantify colony forming units (CFU/mL), serial dilutions of 1 × 10^1^ to 1 × 10^8^ were made in PBS, and the cells were cultured on LB agar for 24 h at 37 °C, as previously described [31].

TCCSUP human bladder cells (ATCC^®^, HTB-5™ cells) were cultured in Eagle’s Minimum Essential Medium (EMEM; ATCC, Manassas, VA, USA) supplemented with nonessential amino acids, 1 mM sodium pyruvate, and 10% fetal bovine serum (FBS, Gibco, MA, USA). The cells (1 × 10^5^) were cultured in 24-well plates and incubated at 37 °C in 5% CO_2_ until they reached an 80% confluent monolayer. The monolayer cells were infected with UPEC strain CFT073 for different times (3 to 5 h) and incubated at 37 °C. At each time point, the supernatants were collected from the wells, centrifuged at 500× *g* for 1 min, and stored at −70 °C for quantification of cytokine levels. A total of 250 µL of trypsin was added to each well containing monolayer cells and bacteria for 7 min, and the reaction was neutralized with 5% FBS. The samples were collected, washed 3 times with PBS, and incubated with 1 mL of 0.1% Triton X-100 for 5 min, and CFU/mL in serially diluted samples (1 × 10^−1^ to 1 × 10^−8^) were determined. All the experiments were conducted in triplicate at three different times. 

### 2.6. Analysis of the Cytokines Production in a Coculture System

The production of pro- and anti-inflammatory cytokines was analyzed in cocultured HTB-5 and HMC-1 cells. Both cell types were incubated in 12-well Transwell^®^ culture plates (Corning^®^ Costar^®^, New York, NY, USA) with a permeable membrane with 4 µm pores and a culture area of 0.33 cm^2^. Briefly, 1 × 10^4^ HMC-1 cells/mL were seeded in the lower chamber of the culture plate, and 9 × 10^4^ HTB-5 cells/mL were seeded in the upper chamber. The cocultured cells were infected with UPEC strain CT073, single mutants (Δ*fimH*, Δ*fliC*, and Δ*csgA*), double mutants (Δ*fimH*Δ*fliC*, Δ*csgA*Δ*fimH*, and Δ*csgA*Δ*fliC*) and previously purified proteins (FimH, FliC, and CsgA) and cultured under the same conditions. At different time points after infection (3 and 5 h), the supernatants of the wells were collected and centrifuged at 500× *g* for 1 min. Cytokine release in the newly generated supernatants was assessed, and the pellet was discarded. PBS and culture media were used as negative controls, UPEC strain CFT073, and purified proteins (FimH, FliC and CsgA) were used as positive controls.

### 2.7. Determination of the Cytokines Levels Using Flow Cytometry

The levels of pro- and anti-inflammatory cytokines, including IL-12, TNF-*α*, IL-10, IL-6, IL-1*β*, and IL-8, were quantified using a BD™ Cytometric Bead Array (CBA) Human Inflammatory Cytokine Kit (Becton, Dickinson Company, BD Biosciences, San Jose, CA, USA) and a BD Bioscience FACSCanto II flow cytometer (BD Biosciences). A mixture of six microbead populations that emitted different fluorescence intensities and were precoated with capture antibodies specific for each cytokine was included in the CBA kit. A total of 50 µL of each sample or coculture supernatant was added to the premixed microbeads in 12 mm × 75 mm Falcon tubes (BD Biosciences). After 50 µL of a mixture of Phycoerythrin -conjugated antibodies) against the different cytokines was added, the mixture was incubated for 3 h in the dark at room temperature. The samples were washed with 1 mL of wash buffer and centrifuged at 500× *g* for 5 min, and the pellet was resuspended in 300 µL of wash buffer. The samples were added to each test tube and analyzed on a FACSCalibur flow cytometer (BD Pharmingen, San Diego, CA, USA) calibrated with setup beads, and 3000 events were acquired for each sample. The data were analyzed with FlowJo 7.6.1 software, and the mean fluorescence intensity was obtained for each sample.

### 2.8. Adherence to HTB-5 Cells

When they reached 80% confluence, monolayer HTB-5 cells (~1 × 10^5^ cells) were cultured in 1 mL of Dulbecco’s modified Eagle’s medium (DMEM; Gibco, Gibco, Thermo Fisher Scientific, Wyman Street, Waltham, MA, USA) and loaded in 24-well plates (Corning^®^ Costar^®^, New York, NY, USA). Briefly, the monolayer HTB-5 cells were infected with 1 × 10^7^ bacteria and cultured for 3 h at 37 °C in a 5% CO_2_ atmosphere. The strains used in this study were cultured in LB medium overnight at 37 °C. The supernatants of the infected monolayer cells were removed, and the attached bacterial cells were gently washed three times with 1 mL of sterile PBS. The infected cells were immediately detached from each well with 1 mL of 0.1% Triton (Amresco Bioscience, Solon, OH, USA) diluted in PBS. The samples were serially diluted (10^−1^ to 10^−5^), and 10 microliters of each sample was cultured on LB agar plates containing the appropriate antibiotic for 24 h at 37 °C. Bacteria attached to the monolayer HTB-5 cells were analyzed quantitatively by determining the CFU/mL in duplicate in two independent experiments.

### 2.9. Statistical Analysis

The data are expressed as the mean and the standard error of the mean (SEM). Statistical analyses were performed using GraphPad Prism 8, and comparisons between groups were made using two-way ANOVA. A value of *p* < 0.05 was considered significant.

## 3. Results

### 3.1. Visualization of Type I Fimbriae, Curli, and Flagella in Different UPEC Strains under TEM

**Figure 1:**
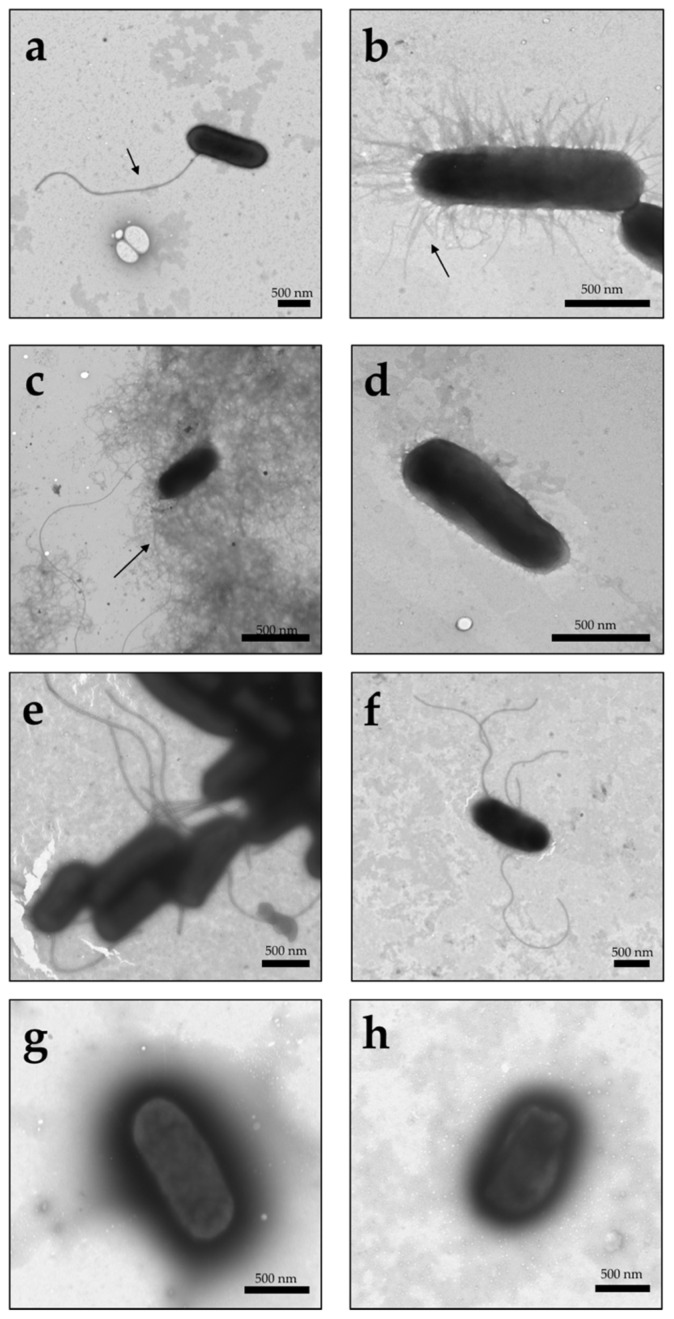
Visualization of type I fimbriae, curli and flagella of the UPEC strain CFT073 by TEM. Bacteria were stained with 1.0% PTA and visualized by TEM. The arrows show (**a**) Flagellum; (**b**) type I fimbriae; (**c**) curli fimbriae. Moreover, (**d**) CFT073Δ*fliC* strain; (**e**) CFT073Δ*fimH* strain; (**f**) CFT073Δ*csgA* strain; (**g**) CFT073Δ*fimH*Δ*fliC* strain; (**h**) CFT073Δ*csgA*Δ*fimH* strain. The scale bars are 500 nm.

To determine the roles of FimH, FliC, and CsgA in adherence and cytokine release, the genes encoding these proteins were disrupted, and single and double mutants were generated. The mutants were confirmed by endpoint PCR and TEM. According to the TEM micrographs, type I fimbriae, curli fimbriae, and flagella were present in UPEC strain CFT073 (Figure 1a–c). The flagella of UPEC strain CFT073 were flexible and large filaments that were approximately 10 µm long and 20 nm in diameter; however, flagellar structures were absent in UPEC strain CFT073Δ*fliC* (Figure 1d).

TEM micrographs also showed the presence of short and rigid structures that assembled in the periphery (peritrichous) of the bacterium, which suggests the presence of type I fimbriae (Figure 1b), in UPEC strain CFT073 under the same nutritional conditions. The TEM micrographs also showed the presence of curli fimbriae, which were visualized as fine coiled fibers, aggregated as an amorphous matrix that extended from 0.5 to 1 mm around the bacterial surface (Figure 1c).

The CFT073Δ*fimH* and CFT073Δ*csgA* strains did not express fimbriae type I and curli fimbriae, respectively, although an increase in the expression of flagella was observed in these mutants (Figure 1e,f). Finally, the strains with double mutations (exemplified by: CFT073Δ*fimH*Δ*fliC* and CFT073Δ*csgA*Δ*fimH*) did not show the presence of flagella, curli, or type I fimbriae (Figure 1g,h).

### 3.2. The Release of Proinflammatory Cytokines Is Induced in a Coculture System

Cocultured cells were infected with UPEC strain CFT073, generated single (CFT073 Δ*fimH*, CFT073Δ*csgA*, and CFT073Δ*fliC*), double mutants (CFT073Δ*fimH*Δ*fliC*, CFT073Δ*csgA*Δ*fliC*, and CFT073Δ*csgA*Δ*fimH*), and purified proteins (FimH, FliC, and CsgA) using the Transwell system in three different ways. Briefly, (1) HTB-5 cells (in the upper chamber) were infected with bacteria, (2) HMC-1 cells (in the lower chamber) were infected with bacteria, and (3) HTB-5/HMC-1 cells (in the upper and lower chambers) were infected with bacteria. The cells were infected for 2, 3, 5, or 6 h, as previously established. The HTB-5 cell viability was decreased by 80% and 90% when cultured at 3 and 5 h, respectively; while the HMC-1 cell viability was decreased by 40% and 80%, when cultured at 3 and 5 h, respectively (data not shown). In this context, the infection assays were performed at 3 and 5 h.

**Figure 2:**
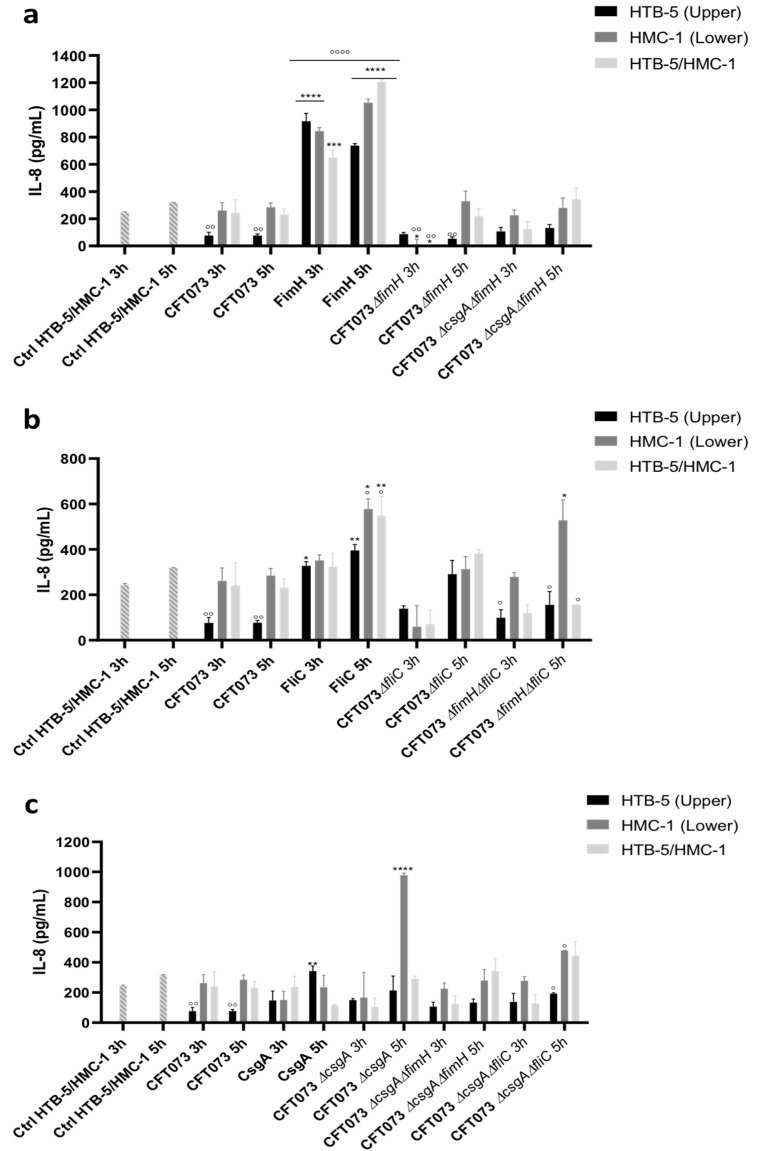
IL-8 release in a coculture system comprising HTB-5 and HMC-1 cells. Cocultured HTB-5 cells (upper compartment), HMC-1 cells (lower compartment), and HTB-5/HMC-1 cells (both compartments) were infected with the reference strain (CFT073), purified protein, strains with mutations in the individual genes or strains with mutations in two genes. (**a**) Cells infected with the CFT073, strains with disruption of the *fimH* gene and the FimH protein; (**b**) cells infected with the CFT073, strains with disruption of the *fliC* gene and the FliC protein; (**c**) cells infected with the CFT073, strains with disruption of the *csgA* gene and the CsgA protein. The mean ± SEM is shown. * *p* < 0.05, ** *p* < 0.005, *** *p* < 0.0005, and **** *p* < 0.0001 compared with cells infected with the reference strain (CFT073). ° *p* < 0.05, °° *p* < 0.005, and °°°° *p* < 0.0001 compared with control cells.

Flow cytometry analysis showed that the concentrations of IL-6 and IL-8 were high; however, the cytokines IL-10, IL-1β, -12p70, and TNF-α were not detectable in any of the established coculture systems. The IL-8 and IL-6 levels in the two cell lines in the coculture system with and without infection with UPEC strain CFT073 were used as reference points for analysis of the infection effects, including with the bacterial strains and purified proteins. IL-8 and IL-6 release was not detected in HTB-5 (upper chamber) and HMC-1 (lower chamber) cells when they were cultured for 3 or 5 h; however, uninfected cocultured cells (HTB-5 cells (upper chamber) and HMC-1 cells (lower chamber) produced basal levels of IL-8 between 245 and 318 pg/mL at 3 and 5 h, respectively (Figure 2).

At 3 and 5 h, a significant decrease in IL-8 release to 76.09 to 76.86 pg/mL was observed in HTB-5 cells infected with UPEC strain CFT073 compared with uninfected cells (*p* < 0.005), infected HMC-1 cells (261.31 and 284.54 pg/mL) and simultaneously infected HTB-5 and HMC-1 cells (240.73 and 231.82 pg/mL (Figure 2). Compared with uninfected cells, HTB-5 cells infected with the CFT073Δ*fimH* strain showed a significant reduction in IL-8 release of 65% (86.67 and 53.42 pg/mL) at 3 and 5 h. Additionally, compared to uninfected cells and UPEC strain CFT073 infected cells, HMC-1 cells infected with the CFT073Δ*fimH* strain for 3 h showed a significant reduction in IL-8 release to 11.52 pg/mL.

Basal IL-8 release was restored in HMC-1 cells at 5 h post-infection. HTB-5/HMC-1 cells simultaneously infected with the CFT073Δ*fimH* strain at 5 h showed a similar pattern of IL-8 release as HMC-1 cells infected with the CFT073Δ*fimH* strain at 3 and 5 h; however, at 3 h after infection, IL-8 release was not restored in HTB-5/HMC-1 cells (Figure 2a).

Under the same conditions, infected cells with the FimH protein showed a pattern of cytokine release inversely proportional to that observed in HTB-5, HMC-1, and HTB-5/HMC-1 cells infected with UPEC CFT073Δ*fimH* strain at 3 and 5 h after infection (Figure 2a). Moreover, 10- and 12-fold increases (737.85 and 916.84 pg/mL) in IL-8 release were observed in HTB-5 cells when infected with FimH at 3 and 5 h, and 3- and 4-fold increases in IL-8 release (844.33 and 1053.16 pg/mL) were observed in HMC-1 cells infected with FimH. Furthermore, 3- and 5-fold increases (649.71 to 1204.27 pg/mL) were observed in HTB-5/HMC-1 cells simultaneously infected with FimH compared with cells infected with the CFT073 and CFT073Δ*fimH* strains. Furthermore, significant differences were observed between the 3 and 5 h post-infection time points (Figure 2a).

Significant changes in IL-8 release were observed in HTB-5 cells infected with the purified FliC protein (327.78 pg/mL at 3 h and 395.66 pg/mL at 5 h), compared HTB-5 cells infected with UPEC strain CFT073 (76.09 mg/mL at 3 h and to 76.86 pg/mL at 5 h). Furthermore, a significant increase in IL-8 release was observed in infected HMC-1 cells (577.95 pg/mL) and simultaneously infected HTB-5/HMC-1 cells (548.95 pg/mL) at 5 h compared with 3 h (351.81 pg/mL and 323.22 pg/mL, respectively); likewise, significant changes between uninfected cocultured and HTB-5/HMC-1 cells infected with UPEC strain CFT073 (Figure 2b). In contrast, HMC-1 cells infected with the CFT073 Δ*fliC* strain showed a significant reduction in IL-8 release at 3 h (351.81 pg/mL) compared with 5 h (577.95 pg/mL); this trend in IL-8 release was similar to that observed for cells infected with the CFT073 strain. Additionally, no significant differences in IL-8 release between infected HMC-1 cells and simultaneously infected HTB-5/HMC-1 cells (Figure 2b). HMC-1 cells infected with the CFT073Δ*fimH*Δ*fliC* strain showed an increase in IL-8 release compared with those infected with the CFT073 strain at 5 h (Figure 2b).

HTB-5 cells infected with CFT037 exhibited reduced IL-8 release compared with uninfected cocultured cells. In contrast, HTB-5 cells infected with purified CsgA protein exhibited a significant increase in IL-8 release at 5 h (342.13 pg/mL) compared with 3 h (Figure 2c). Moreover, compared with uninfected cocultured cells and cells infected with UPEC CFT073 for 3 or 5 h, HMC-1 cells infected with UPEC strain CFT073Δ*csgA* for 5 h showed a significant increase in IL-8 release (977.89 pg/mL). Cells infected with the three mutants showed IL-8 release at 3 h after infection and an increase in the release of this cytokine at 5 h after infection under the three different infection conditions. Compared with uninfected cells, HMC-1 cells infected with UPEC strain CFT073 for 5 h (284.54 pg/mL) and HMC-1 cells infected with UPEC strain CFT073Δ*csgA*Δ*fliC* for 5 h (478.49 pg/mL) showed significant increases in IL-8 release (Figure 2c).

**Figure 3:**
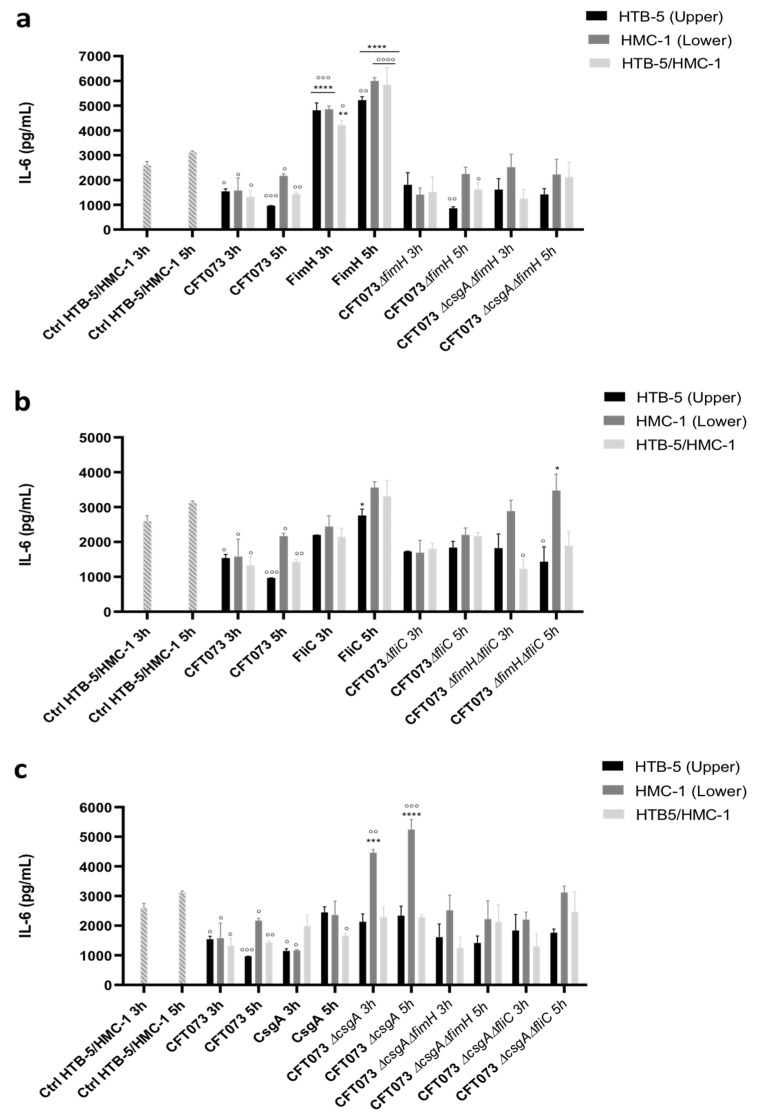
IL-6 release in a coculture system comprising HTB-5 and HMC-1 cells. Cocultured HTB-5 cells (upper compartment), HMC-1 cells (lower compartment), and HTB-5/HMC-1 cells (both compartments) were infected with the reference strain (CFT073), purified protein, strains with mutations in individual genes or strains with mutations in two genes. (**a**) Cells infected with CFT073, strains with disruption of the *fimH* gene, and the FimH protein; (**b**) cells infected with CFT073, strains with disruption of the *fliC* gene, and the FliC protein; (**c**) cells infected with CFT073, strains with disruption of the *csgA* gene, and the CsgA protein. The mean ± SEM is shown. * *p* < 0.05, ** *p* < 0.005, *** *p* <0.0005, and **** *p* < 0.0001 compared with cells infected with the reference strain (CFT073). ° *p* < 0.05, °° *p* < 0.005, °°° *p* < 0.0005, and °°°° *p* < 0.0001 compared with control cells.

Conversely, IL-6 release with uninfected HTB-5/HMC-1 cells produced basal levels of IL-6 between 2602.9 and 3121.87 pg/mL at 3 and 5 h, respectively (Figure 3). Infection with the CFT073 strain under the three infection conditions induced IL-6 release at levels of 1323.58 to 1579.89 pg/mL (at 3 h after infection) and 967.25 to 2170 pg/mL (at 5 h after infection) (Figure 3). The level of IL-6 was significantly increased to 4816.65 pg/mL (at 3 h after infection) and 5223.36 pg/mL (at 5 h after infection) in HTB-5 cells infected with purified FimH protein compared with uninfected cells (2602.9 pg/mL at 3 h after infection and 3121.87 pg/mL at 5 h after infection) and HTB-5 cells infected with UPEC CFT073 (1540.96 pg/mL at 3 h after infection and 967.25 pg/mL at 5 h after infection) (Figure 3a).

Similar levels of IL-6 were observed in HMC-1 cells infected with the FimH protein and HTB-5/HMC-1 cells simultaneously infected with the FimH protein at both time points. HTB-5 cells infected with UPEC CFT073Δ*fimH* did not show significant changes in IL-6 release compared with cells infected with UPEC strain CFT073; however, a significant reduction in IL-6 release was observed in HTB-5 cells infected with UPEC CFT073Δ*fimH* compared to uninfected HTB-5 and HTB-5/HMC-1 cells (Figure 3a). Regardless of the infection site, infection with the purified FliC protein induced a significant increase in IL-6 release at 5 h (between 2760.97 pg/mL and 3562.12 pg/mL) compared to 3 h (2200.06 pg/mL and 2441.95 pg/mL). Infection of cocultured cells with UPEC strain CFT073Δ*fliC* under the three infection conditions did not result in significant changes at 3 or 5 h, or significant differences compared with HMC-1 cells infected with the FliC protein for 3 h (Figure 3b). Additionally, compared with infection for 5 h, infection with purified CsgA protein for 3 h significantly decreased IL-6 levels to 1148.51 pg/mL (HTB-5 cells) and 1169.74 pg/mL (HMC-1 cells). In contrast, HMC-1 cells infected with UPEC strain CFT073Δ*csgA* for 5 h (5242.59 pg/mL) showed a significant increase in IL-6 levels compared with HMC-1 cells infected with this strain for 3 h (4461.35 pg/mL). The double mutants from UPEC strain CFT073 did not cause significant changes in IL-6 release (Figure 3c).

### 3.3. The Roles of the FimH, CsgA, and FliC Genes of UPEC in Adherence to HTB-5 Cells

**Figure 4:**
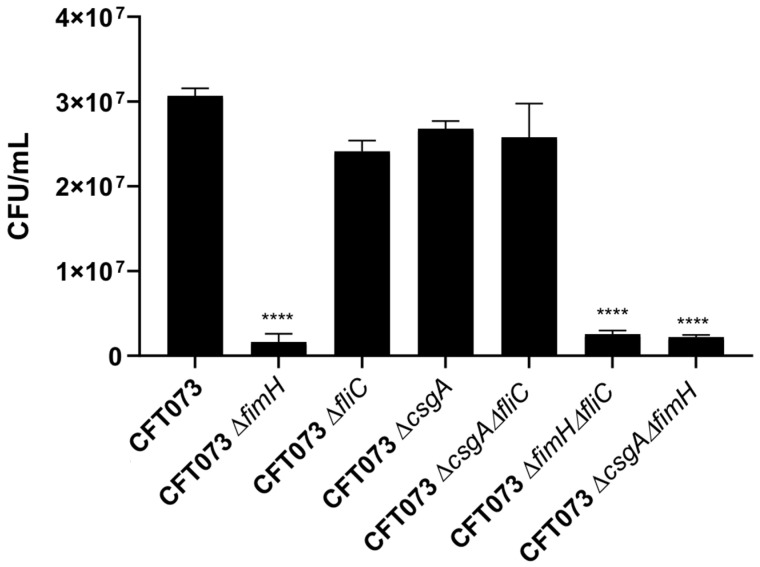
Quantification of adherence to HTB-5 cells. HTB-5 cells were infected with the CFT073 strain or the mutant strains at a MOI of 1:10. The mean ± SEM is shown **** *p* < 0.0001 compared with cells infected with the reference strain (CFT073).

UPEC type I fimbriae, curli fimbriae, and flagella are structures that are assembled in the bacterial periphery and play an important role in the colonization of kidney or bladder cells through specific ligands. To determine the role of the FimH, CsgA, and FliC proteins in bacterial adherence, HTB-5 cells were infected with UPEC strains with mutations in the *fimH*, *csgA*, and *fliC* genes. Quantitative analysis of adherence to HTB-5 cells infected with CFT073Δ*fimH*, CFT073Δ*fimH*Δ*fliC*, and CFT073Δ*csgA*Δ*fimH* revealed significant differences in the adherence percentage to these cells (5.24% (*p* = 0.0001), 6.63% (*p* = 0.0001), and 7.17% (*p* = 0.0001), respectively compared with the adherence percentage to cells infected with UPEC strain CFT073, which was considered 100% (Figure 4). In addition, no difference in the adherence percentage was observed among cells infected with the CFT037Δ*fliC*, CFT073Δ*csgA*, and CFT073Δ*csgA*Δ*fliC* strains. Also, the adherence percentage was similar among the cells infected with the CFT073Δ*fimH*, CFT073Δ*fimH*Δ*fliC*, and CFT073Δ*csgA*Δ*fimH* (Figure 4).

## 4. Discussion

UPEC is the most common etiological agent of complicated and uncomplicated UTIs [4,32,33,34,35,36]. UPEC possess a numerous of virulence factor which gives the bacteria key advantage over its host, between these factors, are capsule, multiple enzymes, and different types of fimbriae, which promote bacterial attachment to the host’s urinary tract tissues [33,34,37]. Type I fimbriae is an essential virulence factor in the colonization of the host by UPEC and is involved in infection of the urinary tract. The fimbriae curli is an accessory molecule used in biofilm development and is considered an adhesin that mediates invasion of the host and induces an immune response. Flagella are mobility structures of UPEC and promote bacterial dissemination toward the upper urinary tract [35]. The host immune response activates various mechanisms to prevent UPEC colonization and survival, such as innate and adaptive responses of the immune system.

The innate immune response is characterized by the production of proinflammatory mediators, including cytokines and chemokines [33]. Two cell lines were used in this study: the TCCSUP ATCC^®^ HTB-5™ and HMC-1 cell lines. HTB-5 cells are from the urinary bladder of a 67-year-old patient diagnosed with grade IV transitional cell carcinoma [38]. HMC-1 cells, which are derived from a patient with mast cell leukemia, have a phenotype similar to that of human mast cells and well-defined phenotypic and genotypic characteristics [39]. HTB-5 human bladder cells and HMC-1 cells present certain morphological characteristics, as described by other authors [39,40]. Our quantitative analysis of cell damage caused by infection showed a decrease in the survival of both cell lines over time. Other studies have reported that mast cells and bladder cells do not exhibit antimicrobial activity against *E. coli* strains [38]. In contrast, HMC-1 cells present anti-pneumococcal cytotoxic activity, and their viability is significantly reduced in the presence of gram-positive pathogens [41,42,43]. Exposure of mast cells to bacteria such as pathogenic *E. coli*, *Streptococcus pneumoniae*, *Pseudomonas aeruginosa*, *Mycoplasma pneumoniae*, and *Mycobacterium tuberculosis* leads to the release of presynthesized and novo synthesized mediators at different times after infection.

The CFT073 strain did not show significant differences in growth when cultured in a nutritional medium or cell culture medium. UPEC successfully adheres to and is internalized in human bladder cells [23]. An alternative to the use of cell monocultures is the use of mixed epithelial cell cultures, also called cocultures, which offer greater flexibility and allow the replication of epithelial barriers and host immune responses. Unlike other culture models, coculture models allow us to obtain information about the interaction between individual cell types [44,45,46]. The objective of this study was to evaluate the release of proinflammatory cytokines in cocultured cells (HTB-5 and HMC-1 cells) induced by infection with UPEC strains (CFT073Δ*fimH*, CFT073Δ*fliC*, CFT073Δ*csgA*, CFT073Δ*fimH*Δ*fliC*, CFT073Δ*csgA*Δ*fimH*, and CFT073Δ*csgA*Δ*fliC*) and purified proteins (FimH, FliC, and CsgA). Only the cytokines IL-8 and IL-6 were detected in the supernatants by flow cytometry. The interaction between bacteria and mast cells and between bacteria and epithelial cells induces the release of many immune response mediators [47]. Our data are consistent with recent studies by our group, which showed that stimulation of HTB-5 cells with UPEC strains results in the release of significant amounts of IL-8 and IL-6 [23].

Tumor necrosis factor (TNF) is responsible for the infiltration of neutrophils, which are key for the resolution of bacterial infections, and is one of the first proinflammatory ILs to be released within the first hour of infection. In addition, UPEC-mediated TNF release occurs 2 h after infection in in vivo models of UTIs but not in in vitro models [47,48]. The release of TNF from mast cells is induced by the release of high concentrations of IL-33 from epithelial cells. IL-33 is released in response to tissue damage, and IL-33 release is induced by IL-37 (cathelicidin), which has a protective function against UTIs since its release is significantly decreased in epithelial cells after infection with UPEC [14,49,50,51,52]. This may explain why TNF was not detected in the coculture model used in this work. IL-1β was also unable to be detected by flow cytometry. Preliminary studies of in vivo models have shown the presence of large amounts of IL-1β; however, the level of IL-1β in HMC-1 cells in vitro is very low [53]. IL-1β is an acute phase IL that is produced early in infection and subsequently stimulates the release of IL-6 and IL-8 in mast cells. The release of IL-1β probably occurs in the first minutes of infection, as reported by other authors [54,55]. IL-12p70 is produced in dendritic cells, macrophages, and neutrophils; however, IL-12p70 release does not occur in HMC-1 cells, which is consistent with what was observed in our study [42,56].

The induction of IL-10 production by UPEC has also been associated with a synergistic interaction between monocytes and uroepithelial cells; however, IL-10 was not detected under the conditions employed in our study [57]. Other studies have shown that IL-10 is produced at 6 h after infection with UPEC in vivo [48]. Recently, UPEC lacking curli fimbriae was described in vivo and was found to induce a significant increase in IL-10 release associated with the expression of the adhesin FimH [23]. Certain cytokines are only expressed in vivo because their release involves simultaneous interactions between a large number of cell populations; this may be the case for IL-10.

Our studies have shown that differences in the levels of IL-8 and IL-6 detected by flow cytometry are related to infection time, strain type, and cell line. Cocultured cells infected with UPEC strain CFT073 showed a significant increase in the release of IL-8 and IL-6; however, smaller amounts of both cytokines were detected in the uninfected cocultured cells. Recently, our group reported that mice infected with curli-producing UPEC strains show a poor release of ILs, probably because the curli fimbriae physically blocks the activity of other fimbriae, which would explain the reduction in IL-6 and IL-8 levels. *Salmonella typhimurium* is capable of suppressing mast cell activation by preventing detection by pattern recognition receptors such as TLR4, suggesting that a bacteria-mediated mechanism delays or completely suppresses host-specific responses [58]. We consider that UPEC can also use this type of mechanism to reduce the secretion of unidentified ILs.

Except for HMC-1 cells infected with CFT073Δ*fimH*Δ*fliC*, cells infected with the others mutant strains with disruption of the *fimH* gene for 3 h showed significantly lower levels of IL-8. Stimulation with the FimH protein induced a significant increase in IL-8 and IL-6 release at 3 and 5 h after infection. The type I fimbriae adhesin FimH is a mannose-binding component and potently stimulates mast cells [47]. CD48 contains mannose, which allows it to easily bind with FimH, and this interaction results in mast cell degranulation [59]. Mast cells regulate the direct attachment of bacteria through TLR2 and TLR4, as well as molecules anchored with glycosylphosphoinositol, i.e., CD48, a membrane receptor for *E. coli* that is expressed on mast cells. Studies have shown that the adhesin FimH also contributes to the release of IL-6 and IL-8 via TLR4 in HTB-5 human bladder cells [25,60].

A significant increase in the release of IL-6 and IL-8 was observed after 3 and 5 h of infection with the purified FliC protein, and a decrease in IL-6 and IL-8 release was observed after 3 h of infection with the strain containing a mutation in the *fliC* gene. Acharya et al. [30] demonstrated that the FliC protein induces the release of IL-10 and other cytokines, including IL-6, via TLR5 in vivo and in vitro. Our data, consistent with other studies, demonstrated that the flagellin FliC stimulates the release of IL-6 and IL-8 in HMC-1 cells. These results show that FliC has an important function but is not essential for the activation of HMC-1 cells and the establishment of an appropriate immune response mediated by the flagellum. The curli fimbria is an amyloid and amorphous structure, and it functions as a physical barrier that prevents other fimbriae from interacting with the uroepithelium surface and therefore blocks the interaction between the uroepithelium surface and TLR4 and consequently the release of IL-6 and IL-8 [25]. In this study, a significant increase in the release of IL-6 and IL-8 was observed in HMC-1 cells infected with the CFT073 Δ*csgA* strain at 5 h. Our data showed that the CsgA protein promotes a significant reduction in IL-8 and IL-6 release, suggesting that its presence prevents the recognition of other fimbriae that could interact with specific ligands.

Compared with infected HTB-5 and HTB-5/HMC-1 cells, HMC-1 cells infected with UPEC strain CFT073 Δ*fimH*Δ*fliC*, which produces the curli fimbriae, showed a significant reduction in IL-6 and IL-8 release. These data support the hypothesis that curli blocks the interaction between other more immunogenic fimbriae and specific ligands located on the cell surface and that its absence results in a more acute proinflammatory immune response against UPEC by the host. However, more studies are required to evaluate the interaction between curli and HMC-1 cells and the host immune response against UPEC as a protective mechanism against damage to the urinary tract. Transwell system infected either in the three different ways with the double mutant strains CFT073Δ*csgA*Δ*fimH* and CFT073Δ*csgA*Δ*fliC* did not show significant production of the proinflammatory cytokines IL-6 and IL-8. In addition, the release of these cytokines by cells infected with the CFT073Δ*csgA*Δ*fimH* strain was similar to that observed in cells infected with FliC, indicating that flagella are involved in the activation of both cell types but suggest are not essential for the initiation an immune response. The release of IL-6 and IL-8 by infected cells with the CFT073Δ*csgA*Δ*fliC* strain was similar to that observed in cells infected with the FimH protein. HMC-1 cells infected with all UPEC strains showed high IL-6 levels. Studies have reported that HMC-1 cells are an important source of IL-6, which promotes mast cell growth through its pleiotropic function [61,62]. Furthermore, in most cases, the release of the cytokines tested in this study was decreased at 3 h after infection and was significantly increased at 5 h after infection. Our data agree with what has been reported in the literature, which suggests that type I fimbriae, curli fimbriae, and flagella are important structures for the pathogenesis of UPEC; however, in the absence of any of these structures, the bacterium regulates the expression of proteins that comprise other structures to restore specific responses to the host [7,58].

Additionally, the adherence of cells infected with the UPEC strains CFT073Δ*fimH*, CFT073Δ*fimH*Δ*fliC*, and CFT073Δ*fimH*Δ*csgA* to HTB-5 cells was significantly reduced, suggesting that type I fimbriae is an essential virulence factor for colonization by UPEC. In contrast, adherence to HTB-5 cells was not significantly altered by inactivation of the *fliC* and *csgA* genes; therefore, curli and flagella are important but not essential accessory structures of UPEC in this model of adherence. Our data suggest that curli mainly regulates the specific immune response of the host by significantly decreasing it and is a colonization factor that contributes to adherence and epithelial damage. By reducing the host’s immune response, the curli fimbriae allow more efficient replication of the bacterium, and its expression may be related to the persistence of UPEC in the urinary tract. In conclusion, type I fimbriae, curli fimbriae, and flagella are involved in the release of IL-6 and IL-8 by cocultured HTB-5 and HMC-1 cells at 3 and 5 h after infection.

## Figures and Tables

**Table 1:** The UPEC strains and plasmids used in this study.

Bacterial Strain and Plasmid.	Features	Reference
Bacterial Strains		
CFT073	UPEC Human clinical specimen: blood and urine from a woman with acute pyelonephritis.	ATCC
CFT073Δ*fimH*	*fimH* disruption, Km^R^	Luna-Pineda et al. [25]
CFT073Δ*fliC*	*fliC* disruption, Km^R^	This study
CFT073Δ*csgA*	*csgA* disruption, Km^R^	Luna-Pineda et al. [25]
CFT073Δ*fimH*Δ*fliC*	*fimH* and *fliC* disruption, Km^R^ Cm^R^	This study
CFT073Δ*csgA*Δ*fimH*	*csgA* and *fimH* disruption, Km^R^ Cm^R^	Luna-Pineda et al. [25]
CFT073Δ*csgA*Δ*fliC*	*csgA* and *fliC* disruption, Km^R^, Cm^R^	This study
Plasmids		
pKD46	Plasmid expressing the λ phage recombination system (pBAD-λ-Red (γ β exo) Ap^R^	Datsenko and Wanner [31]
pKD4	Template vector for amplifying FRT-kan FRT; *bla* FRT *km* P1 P2 oriR6K Km^R^	Datsenko and Wanner [31]
pKD3	Template vector for amplifying the *cat, bla* FRT cm P1 P2 oriR6K Cm^R^ gene	Datsenko and Wanner [31]

**Table 2:** Primers used for inactivation of the *fliC*, *fimH,* and *csgA* genes in UPEC strain CFT073.

Primer	Sequence 5′–3′	Resistance Cassette	Product Size (bp)
*fliCm*-F	ATGACGCCGCGGGTCAGGCGATTGCTAACCGTTTTACTTCTAACATTAAAGGCCTGACTCGTGTAGGCTGGAGCTGCTTC	pKD3 (Cm^R^)	1300
*fliCm*-R	TCTGCGCTTTCGACATGTTGGACACTTCGGTCGCATAGTCGGCGTCCTGAATACGGGACTCATATGAATATCCTCCTTAG	pKD4 (Km^R^)	1600
*fimHm*-F	TATACCTACAGCTGAACCCAAAGAGATGATTGTAATGAAACGAGTTATTAGTGTAGGCTGGAGCTGCTTC	pKD3 (Cm^R^)	1300
*fimHm*-R	CCTGCATTAGCAATGCCCTGTGATTTCTTTATTGATAAACAAAAGTCACGCCCATATGAATATCCTCCTTAG	pKD4 (Km^R^)	1800
*csgAm*-F	GTTTTACATGAAACTTTTAAAAGTAGCAGCAATTGCAGCAATCGTATTCGTGTAGGCTGGAGCTGCTTC	pKD3 (Cm^R^)	1300
*csgAm*-R	GCGCCCTGTTTCTTTCATACTGATGATGTATTAGTACTGATGAGCGGTCGCATATGAATATCCTCCTTAG	pKD4 (Km^R^)	1800

**Table 3:** Primers used to verify the inactivation of the *fliC*, *fimH*, and *csgA* genes in UPEC strain CFT073.

Primer	Sequence 5′–3′	Length	GC Content (%)	Tm (°C)	Product Size (bp)
*fliCv*-F	GGATCCCAGACGATAACAGGGTTGACGGC	29	58.6	65.2	1923
*fliCv*-R	GAGCTCTCAGGCAATTTGGCGTTGCCGTC	29	58.6	65.2	
*fimHv*-F	GAGCTACAGGATGACAGTGGC	21	57.1	57.5	1237
*fimHv*-R	GGAACAGACCAGCAAAGTGC	20	55	56.8	
*csgAv*-F	GCCAGTATTTCGCAAGGTGC	20	55	57.1	789
*csgAv*-R	GGTGTACATATCCCCTTGCTGG	22	54.5	57.4	

## Data Availability

All relevant data are provided in the manuscript.
